# MicroRNAs as Molecular Biomarkers for the Characterization of Basal-like Breast Tumor Subtype

**DOI:** 10.3390/biomedicines11113007

**Published:** 2023-11-09

**Authors:** Muhammad Tariq, Vinitha Richard, Michael J. Kerin

**Affiliations:** Discipline of Surgery, Lambe Institute for Translational Research, H91 TK33 Galway, Ireland; m.tariq1@universityofgalway.ie

**Keywords:** basal-like breast cancer, hormone receptors, classification, microRNAs, transcriptomics, triple-negative breast cancer

## Abstract

Breast cancer is a heterogeneous disease highlighted by the presence of multiple tumor variants and the basal-like breast cancer (BLBC) is considered to be the most aggressive variant with limited therapeutics and a poor prognosis. Though the absence of detectable protein and hormonal receptors as biomarkers hinders early detection, the integration of genomic and transcriptomic profiling led to the identification of additional variants in BLBC. The high-throughput analysis of tissue-specific micro-ribonucleic acids (microRNAs/miRNAs) that are deemed to have a significant role in the development of breast cancer also displayed distinct expression profiles in each subtype of breast cancer and thus emerged to be a robust approach for the precise characterization of the BLBC subtypes. The classification schematic of breast cancer is still a fluid entity that continues to evolve alongside technological advancement, and the transcriptomic profiling of tissue-specific microRNAs is projected to aid in the substratification and diagnosis of the BLBC tumor subtype. In this review, we summarize the current knowledge on breast tumor classification, aim to collect comprehensive evidence based on the microRNA expression profiles, and explore their potential as prospective biomarkers of BLBC.

## 1. Introduction

Breast cancer is the most commonly diagnosed malignancy in women globally with over 2 million new cases diagnosed in 2020 [1]. The American Cancer Society projected 300,590 new cases of breast cancer estimated to account for 43,700 deaths in the United States alone in 2023 [2]. Breast cancer is a highly heterogeneous disease and can be further categorized based on various parameters including and not limited to location of origin, degree of cellular pleomorphism, molecular composition, etc. [3,4]. The classification of breast tumors based on the expression of certain hormone receptors, analyzed via immunohistochemistry (IHC) is a conventionally recognized method [5]. The hormone receptors used are the following: estrogen receptor (ER), progesterone receptor (PR), and human epidermal growth factor receptor 2 (HER2) [5,6]. Based on the expression of the aforementioned receptors, four subtypes of breast cancer are widely recognized and are as follows: luminal A, luminal B, HER2+, and triple-negative breast cancer (TNBC) [7]. The triple-negative breast tumor subtype lack the expression of ER, PR, or HER2 receptors and account for 10–15% of total breast cancer cases [8]. TNBC records a high recurrence rate with an inferior prognosis relative to non-TNBC [9]. The prognosis and treatment regimen for breast cancer can differ significantly from patient to patient owing to the substantial heterogeneity between primary malignant tumors [10].

The term “triple-negative breast cancer” is often used synonymously with “basal-like breast cancer”, and although the two possess a degree of resemblance to one another, they are not entirely interchangeable terms [11]. The exact definition of basal-like breast cancer (BLBC) is a topic of debate, but the title was initially designated to tumors characterized by an expression of markers for basal epithelial cells including cytokeratins (CK) 5/6 and CK17 with low or negative ER expression [12]. TNBCs and BLBCs are both associated with higher grade disease, larger size tumors, and aggressive histopathologic characteristics [13]. Individuals with BLBCs have decreased overall survival, with a decreased disease-free period alongside a statistically significant increased risk of recurrence post-chemotherapy relative to other subtypes [14]. Considering the aggressive nature and disease process of BLBCs, there still exists a gap in the therapeutic options for the pathology and, as of yet, chemotherapy remains the mainstay [15]. Therefore, early disease detection and the development of targeted treatment are imperative to reducing death rates and improving BLBC patient survival outcomes and prognosis [16].

Researchers have worked to segregate breast cancers in many ways, including the IHC-mediated proteomic approach of classifying tumors based on hormone receptor expression as previously mentioned [7]. The genome-based partition of breast cancer is another potential approach, and a well-accepted genomic design is the 50-gene microarray panel PAM50 (prediction analysis of microarray 50) [17]. A study by Krug et al. highlights a proteogenomic technique that may be used to identify clinical and molecular features of specific tumors for the development of tumor-specific treatment targets [18]. Transcriptomic characterization of malignant breast tumors based on microRNAs is a novel approach currently under ongoing investigation [19,20,21,22].

MicroRNAs are established post-transcriptional modulators of gene and consequentially protein expression that play a pivotal role in the maintenance of bodily homeostasis and the evolution of various pathological processes [23,24]. MicroRNAs have the ability to travel extracellularly in blood and plasma, prompting them to be the ideal subject for the development of a non-invasive molecular-based system for precise identification and classification [22,25]. This review aims to explore the use of a miRNA-based model to diagnose, subclassify, predict treatment response, evaluate the prognosis, and formulate potential targeted therapeutics for the BLBC subtype.

## 2. Evolution of the BLBC Classification System

From the initial four subtype classification system based on IHC data, breast cancer subtype classification has continued to develop throughout the years. Various other panels have been suggested to specifically identify BLBCs, one of which was introduced by Nielsen et al. that claims an IHC-based panel of ER, human epidermal growth factor receptor 1 (HER1), HER2, and cytokeratin (CK) 5/6 can distinguish BLBC tumors with high specificity [26]. Cheang et al. introduced a similar expanded immunopanel that consisted of ER, PR, HER2, EGFR, and CK5/6 in order to implement a precise definition of BLBC [27]. Livasy et al. found that BLBC tumors most coherently exhibited an immunophenotype of negative ER and HER2 expression in addition to CK5/6, CK8/18, epidermal growth factor receptor (EGFR), and vimentin positivity [28]. In 2007, Herschkowitz et al. discovered a subtype of breast cancer and referred to this as “claudin-low” tumors that are characterized by low expression of claudins 3, 4, 7, occludin, and E-cadherin [29]. Prat et al. then characterized the “claudin-low” subtype phenotypically and molecularly and found it to be ER negative [ER−], PR negative [PR−], and HER2 negative [HER2−], but showed an inconsistent expression of basal cytokeratins [30]. Claudin-low tumors were found to display phenotypic and molecular characteristics shared between the basal-like and luminal subtypes, henceforth bringing upon the emergence of another intrinsic subtype of breast cancer [30].

Parker JS introduced a 50-gene centroid-based breast cancer subtype identification model to compare the reproducibility of subtype assignment through the use of a prediction analysis of microarray (PAM) algorithm termed PAM50 [17]. They used Spearman’s rank correlation to identify the nearest simple centroid, which was then assigned as one of the following molecular intrinsic subtypes: luminal A, luminal B, HER2-enriched, basal-like, and normal-like [17,31]. The PAM50 gene microarray panel has been shown to subclassify breast cancer and the risk of relapse (ROR) with a high degree of reproducibility and validity, making it a commonly implemented system [17,32].

**Figure 1:**
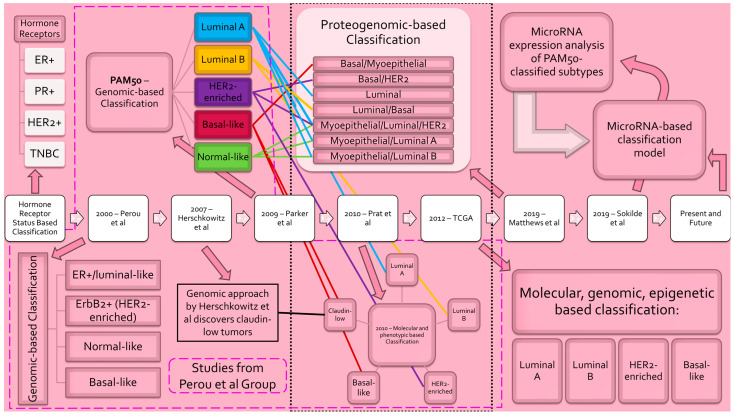
Evolution of breast cancer subclassification. Progression of breast cancer molecular subtyping schema [12,17,19,29,30,33,34]. Classification models are listed in chronological order from left to right. Blue lines denote correspondence to PAM50 luminal A subtype. Yellow lines denote correspondence to PAM50 luminal B subtype. Purple lines denote correspondence to PAM50 HER2-enriched subtype. Red lines denote correspondence to PAM50 basal-like subtype. Green lines denote correspondence to PAM50 normal-like subtype. _____ denotes correspondence to Herschkowitz et al.’s claudin-low subtype. - - - - encompasses studies completed by the Perou et al. group. ......... denotes classification model incorporating PAM50. [Abbreviations: ER—estrogen receptor; PR—progesterone receptor; HER2—human epidermal growth factor receptor 2; TNBC—triple-negative breast cancer; ErbB2—avian erythroblastic leukemia viral oncogene homolog 2; PAM50—prediction analysis of microarray 50].

The Cancer Genome Atlas (TCGA) project incorporated molecular data of 825 breast cancer patient samples across five technological platforms and rendered four distinct heterogeneous breast cancer subtypes: luminal A, luminal B, HER2-enriched, and basal-like [33]. A novel breast cancer subtype classification system based on messenger RNA (mRNA) signatures incorporated with the PAM50 50-gene signatures identified seven breast cancer subtypes with clearly defined groups of gene expression patterns [34]. The distinct taxonomic groups proposed are as follows: basal/HER2, basal/myoepithelial, myoepithelial/luminal/HER2, myoepithelial/luminal, luminal, basal/luminal, and basal/luminal/HER2 [34]. A phenogenomic analysis of 483 breast cancer tumors by Ali et al. incorporated single-cell phenotypical analysis alongside genomic profiling and rendered their own distinct subgroups, which is a testament to the ongoing progression of breast tumor subclassification [35]. New microRNA-based panels may yield a more efficient, uniform, and clinically pertinent BLBC signature for the identification and prognostic indication and are in continued development [22,36]. To that point, Søkilde R et al. conducted transcriptomic analysis and sequencing of miRNAs on 186 PAM50 classified breast cancer samples from the Sweden Cancerome Analysis Network–Breast (SCAN-B) trials and categorized breast cancer based on the miRNA expression profiles [19,37]. The work conducted by Søkilde R et al. has paved the way for future validation studies and the progression of miRNA-based breast cancer research (Figure 1).

## 3. Significance of MicroRNAs in Breast Tumor Pathogenesis

### 3.1. Biogenesis of MicroRNAs

MicroRNAs (miRNAs) are small non-coding RNA molecules, typically 21–24 nucleotides in length, that are endogenously expressed and play a crucial role in post-transcriptional gene expression regulation, leading to variations in protein expression levels [38,39]. In the human genome, genes encoding miRNAs are predominantly located within intronic sequences, with a minority originating from exons or as intergenic sequences, each possessing its own promoter [40]. MicroRNAs undergo a well-planned series of events throughout their formation [41].

The transcription of miRNA genes yields primary miRNAs (pri-miRNAs) that undergo a two-step cleavage process, ultimately forming mature miRNA [41]. The process begins with RNA Polymerase II (RNA Pol II) transcription of the miRNA genes, which is followed by nuclear processing made possible by the Drosha ribonuclease III (DROSHA)-DiGeorge syndrome critical region 8 (DGCR8) complex [42]. The protein DGCR8, which binds to double-stranded RNA, aids in identifying the stem-loop structure present in pri-miRNAs [42]. Pre-miRNAs are shorter hairpin-shaped RNA molecules that are produced as a result of the ribonuclease DROSHA cleaving the pri-miRNA at a particular point inside the stem loop [43]. Exportin-5 (XPO5) then transports these pre-miRNAs from the nucleus to the cytoplasm [44]. These mature miRNAs integrate into the RNA-induced silencing complex (RISC), a key effector complex [45]. MiRNAs exhibit versatile modes of action, including binding to the 3′ untranslated region (UTR) of target mRNAs to repress translation and induce mRNA destabilization [46,47]. Additionally, they can bind to the 5′ UTR, resulting in gene expression silencing, or directly interact with promoter regions of protein-coding genes, eliciting transcription [40,48]. Remarkably, a single miRNA has the potential to target hundreds of distinct mRNAs, rendering the overall impact of miRNAs on gene regulation highly intricate [49].

### 3.2. The Dual Regulatory Roles of MicroRNAs in Cancer

MiRNAs play pivotal roles in various biological processes, including nervous system regulation, cell differentiation, development, viral infection, angiogenesis, cancer, gastrointestinal diseases, and diabetes [50]. Eugene Makeyev and Tom Maniatis enrich our understanding of miRNA versatility and also offer a compelling explanation for the surprisingly minimal disparities in protein-encoding genes across organisms of varying morphological and behavioral complexities, emphasizing the pivotal role miRNAs play in shaping intricate gene regulatory networks during cellular differentiation [51]. The evolution of cancer within an individual is the product of the malignant cells and the extrinsic factors interacting with and potentially dictating the behavior of said cells. The hallmarks of cancer (tumor-promoting inflammation, inducing or accessing vasculature, genome instability and mutation, resisting cell death, etc.) may all be possible targets of miRNAs [52]. MicroRNA dysregulation can result from multiple mechanisms, including gene locus expansion inducing gene overexpression, mutations leading to loss of function or deregulation, epigenetic modifications influencing gene activation or suppression, and trans-acting elements on gene expression control [53].

Studies have found that miRNAs function by altering the translation/transcription of oncogenes and/or tumor suppressor genes (TSGs) [54]. Oncogenic miRNAs (oncomiRs) tend to be overexpressed in cancer with the inverse relationship being true for tumor-suppressive miRNAs [49]. Disruption of miRNA regulation may wield significant influence over cancer progression, and an example of such is the oncomiR miR-21 which has been shown to drive tumor invasion, metastasis, and create epithelial mesenchymal transition (EMT) [55]. MiRNAs may also operate to antagonize or downregulate oncogenes (anti-oncomiR) and TSGs, further highlighting their role as modulators of carcinogenesis, cell apoptosis, invasive potential, and treatment resistance or efficacy [56]. The miRNA expression profile of specific tumors is the subject of ongoing investigation as a means of potential treatment avenue, early detection, or sub-classification.

The C19 miRNA cluster (C19MC) is the most tightly co-expressed miRNA set among all miRNAs that are expressed and a high expression of C19MC miRNAs also marked the basal-like TNBC subtype [57]. Similarly, the miR-17-92 cluster exhibits oncogenic behavior and has been observed to show substantial overexpression in human lung cancer and also contributes to the oncogenesis of acute myeloid leukemia with mixed-lineage leukemia (MLL) rearrangement [58]. The elevated amplification of the miR-23a locus, in conjunction with other miRNAs, has been found to stimulate gastric cancer tumor growth while simultaneously diminishing the expression of the metallothionein 2A (*MT2A*) gene, a known tumor progression suppressor [59,60]. In primary breast cancer cells, forced expression of miR-191 and miR-425 was shown to suppress the expression of dicer 1, ribonuclease III (*DICER1)* post-transcriptionally, consequently leading to the advancement of tumor progression and metastasis [61]. Repression of miR-191/425 was seen to also repress the carcinogenic activity of primary breast cancer cells, implicating miR-191/425 as oncomiRs [61]. MiRNAs such as miR-520h have been found to exhibit a dualistic role in cancer, alternating between tumor-suppressing and tumor-promoting functions [62]. In contrast, miR-15a and miR-16-1 consistently act as potent tumor suppressors, particularly in chronic lymphocytic leukemia, where they target the anti-apoptotic B-cell lymphoma 2 (*BCL-2*) mRNA [63]. Alternatively, miR-29 serves as a tumor suppressor in cholangiocarcinoma by targeting myeloid leukemia 1 (MCL-1) and in acute myeloid leukemia by orchestrating DNA methyltransferases, thereby restoring the expression of tumor suppressor genes through DNA hypomethylation [64].

Despite their ability to affect a wide range of target genes, individual miRNAs frequently exert their physiological effects by subtly altering the concentrations of crucial cellular proteins that serve as essential elements within complex cellular signaling pathways [65]. In hormone receptor positive breast cancer, the concentration of circulating miR-195 and let-7a was significantly elevated and was observed to discern breast cancer patients with a specificity of 100% for both and a sensitivity of 85.5% for miR-195 and 77.6% for let-7a [66]. In TNBC, hsa-miR-29b-3p has been found to elicit oncogenic activity through modulation of tumor necrosis factor receptor associated factor 3 (TRAF3), while miR-29a was found to downregulate the TSG suppressor of variegation 4–20 homolog 2 (*SUV420H2*) [47,67,68]. In summary, miRNAs operate to inflect the physiological, pathophysiological, and pathological processes within the body and are subject to continued research for the identification of their exact functional and pathological mechanism of action [69] (Table 1).

## 4. MicroRNAs in Basal-like Breast Cancer

### 4.1. MicroRNAs in the Diagnosis and Substratification of BLBC/TNBC Subtype

Due to the aggressive nature of basal-like breast cancers (BLBCs) and triple-negative breast cancers (TNBCs), early detection and diagnosis are imperative [83]. A screening assay or miRNA signature panel that differentiates BLBCs or TNBCs from the other subtypes or normal tissue may allow for quicker diagnosis and earlier intervention [56]. An example of such is the seven-miRNA panel developed by Kahraman et al. that was able to distinguish healthy women from patients with basal-like TNBC with a sensitivity and specificity of 84% and 74%, respectively [84]. Illustrating the miRNA profile of BLBCs/TNBCs can pave the way to understanding its meticulous disease process and lead to future studies validating the distinct role of miRNAs on tumorigenesis and tumor suppression [85,86].

One avenue to address this is through the analysis of miRNAs over- and under-expressed in TNBC and one such study was conducted by Hu J et al. [87]. The expression of seven miRNAs (hsa-miR-93, hsa-miR-25, hsa-miR-328, hsa-miR-16-2, hsa-miR-BART6-3p, hsa-miR-106b, and hsa-miR-7) were found to be upregulated by more than two-fold, while the expression of ten miRNAs (hsa-miR-145, hsa-miR-205, hsa-miR-27a, hsa-miR-195, hsvl-miR-H8, hsa-miR-376c, hsa-Let-7a, hsa-miR-585, hsa-miR-144, and hsa-miR-371-5p) were found to be downregulated [87]. A similar study by Radojicic J et al., using quantitative polymerase chain reaction (qPCR), observed significant overexpression of miR-21, miR-210, and miR-221 with reciprocal under expression of miR-10b, miR-122a, miR-145, and miR-205 in an analysis of 49 primary TNBC samples [88]. Ouyang M et al. analyzed the expression of 1513 miRNAs in TNBCs and found 41 miRNAs to be significantly dysregulated, 18 upregulated, and 23 downregulated, relative to normal adjacent tissue samples [89]. Du Y et al. performed a similar investigation of miRNAs differentially expressed in TNBC relative to normal, and found miR-105-5p, miR-210-3p, and miR-767-5p to be specifically upregulated with reciprocal under expression of miR-5683 [90]. Søkilde R et al.’s miRNA sequencing analysis of PAM50-classified BLBC samples found over one thousand miRNAs differentially over- and under-expressed in basal-like samples [19]. Similar studies on miRNAs up- and downregulated in BLBC/TNBC are featured in Table 2.

### 4.2. MicroRNAs Identified as OncomiRs or Tumor Suppressors in BLBC/TNBC

MiRNA expression signatures have been implicated as a means for the development of a potentially effective screening assay for the detection of breast cancer and its subtypes via a liquid blood/plasma-based biopsy sample [96]. In order for such a panel to be constructed, the identification of miRNAs that play a role in BLBC/TNBC and their specific functions and targets is imperative. Overexpression of miR-20a-5p was found to downregulate the expression of *Bim* and *p21*, in turn promoting metastasis and invasiveness of TNBC tumor cells in vitro, implicating miR-20a-5p as an oncomiR for TNBC [97]. In a miRnome analysis of miR-135b expression, it was found to be exclusively related to TNBC with basal-like phenotype and identified to possess carcinogenic activity [98]. Further target mapping of miR-135b implied its mechanism of tumorigenesis was through modulation of the wingless-related integration site (*WNT),* transforming growth factor-beta (*TGFβ)*, and erythroblastic leukemia viral oncogene homologue (*ERBB)* pathways, all of which have been associated with breast cancer oncogenesis [98,99,100,101]. Hu et al. found miR-93 to be overexpressed in TNBCs relative to non-TNBCs and found MCF-7 cells transfected with miR-93 plasmid (pS-miR-93) displayed increased proliferation and invasion relative to the control [87]. The Warburg effect, a surging hallmark of cancer and a phenomenon in which malignant cells undergo increased aerobic glycolysis was found to be heavily correlated to miR-210-3p, miR-105-5p, and miR-767-5p in TNBC [90].

The stem-cell factors sex-determining region Y-related high-mobility group box 9 (*SOX9*) and aldehyde dehydrogenase 1 (*ALDH1*), the most activated factors in ductal carcinoma in situ (DCIS) stem-like cells, were identified to be objective targets for miR-140 [102]. The expression levels of miR-140 were seen to be downregulated in cancer stem-like cells relative to normal, and the reinstatement of miR-140 expression was shown to reduce in vivo tumor progression and exhibit tumor suppressive function in basal-like DCIS [102]. Telomeric repeat-binding factor 2 (*TRF2*), a known cancer advancement contributor, regulates telomere maintenance and has been identified to be downregulated by miR-182-3p in TNBC [103,104]. Impaired TNBC tumor expansion was seen in models treated with lipid nanoparticles containing miR-182-3p within [104]. Further miRNAs identified as oncomiRs and tumor suppressors in BLBC and/or TNBC summarized with their validated target and regulated expression can be found in Table 3.

### 4.3. Role of MicroRNAs as Prognostic Indicators in BLBC/TNBC

Prognostic indicators may be used to estimate the probability of crucial parameters such as overall survival (OS), recurrence-free survival (RFS), response to specific treatment regimens, and metastasis [123]. MiRNAs are a prime candidate for future enrichment and validation studies into their potential prognostic implications as their function is quite nuanced and not entirely defined [85,124]. Previous literature exploring the prognostic connotation of miRNA expression signatures has already shown great promise, and some such studies are showcased in Table 4.

## 5. MicroRNAs in Modulation of Drug Resistance in Breast Cancer

MiRNAs also play a pivotal role in modulating drug resistance in cancer cells and an example of such is miR-519c which was identified as a key factor in enhancing drug sensitivity in colon cancer cells through its regulation of ATP-binding cassette super-family G member 2 (ABCG2) [129]. Notably, miR-21 inhibition proves efficacious in reducing topotecan resistance in breast cancer [130]. The functional dynamics of miR-451 in doxorubicin-resistant breast cancer cells are nuanced, as it can either enhance sensitivity to anticancer drugs or provide protection [131]. Doxorubicin-resistant breast cancer tumors display suppressed or decreased expression of miR-451 and miR-200c and the transfection of miR-451 and miR-200c to MCF-7/doxorubicin-resistant cells elicits a heightened sensitivity to doxorubicin in said cells [131,132,133]. The heightened sensitivity underscores the critical role of correcting miRNA expression anomalies in the development of effective therapeutic approaches against drug-resistant cancer cells [131,132,133].

Beyond doxorubicin, other chemotherapeutics have also harnessed the potential of miRNAs to combat breast cancer [134]. Another such example is that of miR-328 demonstrating the capacity to enhance cellular sensitivity to mitoxantrone by downregulating the expression of ABCG2 proteins, resulting in an increased susceptibility of malignant tissue to mitoxantrone [134,135]. Furthermore, miR-132-3p enhances the susceptibility of breast cancer cells to etoposide-induced apoptosis, thereby increasing their responsiveness to the cytotoxic effects of etoposide [134,136]. Hu et al. highlighted the crucial role of miRNA-452 in influencing the responsiveness of breast cancer cells to docetaxel (DOC) where elevated miRNA-452 levels reduced sensitivity to DOC treatment, and conversely decreased miRNA-452 levels enhanced drug sensitivity [134,137]. Additionally, an analysis of miRNA and mRNA expression patterns identified a group of miRNAs, including let-7i, miR-346, miR-638, miR-181a, miR-191, miR-199b, miR-204, miR-211, miR-212, miR-216, miR-328, miR-373, miR-424, miR-768-3p, and miR-221/222, whose reduced levels significantly contributed to the development of antiestrogen resistance in breast cancer, presenting potential avenues for improved therapeutic strategies aimed at addressing this clinical challenge [134,138]. Identifying the role of miRNAs in determining iatrogenic complications of chemotherapy has significant clinical implications for patient satisfaction and prognosis [139,140]. Decreased concentration of circulating miR-195 and miR-21 was found to be statistically significant in forecasting the development of neutropenia and mucositis, respectively, in patients with primary breast cancer receiving neoadjuvant chemotherapy [140]. In the same study, elevated concentrations of miR-10b were found to correlate well to the development of anemia while raised circulating miR-145 expression accurately identified patients encountering nausea and vomiting [140].

## 6. MicroRNAs as Potential Treatment Options for Breast Cancer

Novel targeted treatment has already shown promising results and is well highlighted in a phase 3 trial of adjuvant pembrolizumab, an antiprogrammed death 1 checkpoint inhibitor, immunotherapy alongside neoadjuvant chemotherapy which showed increased rates of pathological complete response (pCR) and survival in early TNBCs [141]. Through functional mapping of miRNAs, therapeutics targeting miRNAs can be developed and instilled to improve the prognosis of BLBC and TNBC [142]. miR-10b exhibits therapeutic potential by targeting the homeobox D10 (*HOXD10*) gene, utilizing an Antagomir delivery system via the pcDNA5-CMV-d2eGFP vector [143]. MiR-19a-3p holds promise through its modulation of the fos-related antigen 1 (*FRA-1*) proto-oncogene, employing nanoparticles as the delivery platform within the category of miRNA mimics [144]. Furthermore, miRNAs such as miR-27a and miR-451 have demonstrated therapeutic relevance by targeting the multidrug resistance protein 1 (*MDR1*)*/P-glycoprotein* gene, facilitated through lipid-based delivery systems, categorized as miRNA mimics/antagomirs [145]. MiR-34a, on the other hand, exerts its influence by targeting multiple genes, including E2F transcription factor 3 (*E2F3*), cluster of differentiation 44 (*CD44*)*,* and silent mating-type information regulation 2 homolog 1 (*SIRT1*), by employing the T-VISA system (plasmid) within the miRNA mimics category [146]. MiR-145 showcases its therapeutic potential through the regulation of various genes, including fascin-1 (*FSCN1*), *C-MYC*, small mothers against decapentaplegic homolog 2/3 (*SMAD2/3*), insulin-like growth factor 1 receptor (*IGF-1R*), and tumor protein 53 (TP53), utilizing adenoviral and lentiviral vectors as the transfection systems in the miRNA mimics category [147,148]. MiR-326 takes aim at the multidrug resistance protein 1 (MRP-1)/ATP-binding cassette subfamily member 1 (*ABCC1*) gene, employing the pGL2-control vector for transfection [149]. Additionally, miR-298 and miR-1253 demonstrate their therapeutic relevance by targeting the *MDR1/P-glycoprotein* (P-gp) gene, making use of the lipofectamine vector in the miRNA mimics category [150]. Even though it is a promising field for the ongoing battle against cancer, it is not without its limitations which include challenges with reproducibility, delivery of therapeutics, knowledge gaps, and cost efficiency [151,152,153,154].

## 7. Limitations and Challenges of MicroRNA-Based Research

The implication of miRNAs in cancer is a relatively recent concept, evidenced by the fact that the first study showing dysregulation of miRNAs in cancer was published as recently as 2002, which suggested the role miR-15 and miR-16 in chronic lymphocytic leukemia [155]. Validation studies have shown inconsistent results, which may be a consequence of variation in methodology and lack of a standardized approach [151,153]. Disparities in the miRNA quantification method used such as hybridization, amplification, and sequencing can directly contribute to sub-satisfactory reproducibility [153]. Discrepancy of sample extraction and preparation, miRNA retrieval, miRNA quantitative and qualitative protocols, and normalization of post-analytical data all may give rise to conflicting findings [151]. Clinical implementation of miRNA-based medications faces significant obstacles including identifying viable mediums for administration, impaired transfection, adverse side effects, and bioavailability/biodistribution, as well as navigating patient-specific discrepancies in metabolism [155]. These challenges are exemplified due to the apparent inadequate understanding of the complete functionality for any given miRNA, and the in vivo antagonization or introduction of one or more miRNAs possesses the capacity to generate considerable drug-associated toxicity [154,155]. This is well evidenced from previous clinical trials involving miRNA interference drugs; an example of such is the miR-34 mimic, MRX34, which showed substantial efficacy against primary liver cancer, melanoma, renal cell carcinoma, and non-small cell lung cancer but induced five significant immune-related adverse events leading to trial termination [154]. Cost optimization without compromising integrity and quality also poses as a hinderance to miRNA-based research [154]. Investigations to surmount the above-mentioned impediments, to facilitate the clinical translation of miRNAs, are ongoing and promising [156].

## 8. Conclusions

Breast cancer is an ever-prevalent pathology that persistently claims lives by the thousands yearly. Researchers have investigated the complex, heterogenous disease process of breast cancer to taxonomically segregate tumors based on their proteomic, genomic, phenotypic, transcriptomic, and epigenetic profile. Of all the resultant variants, BLBC and TNBC carry an unfortunate prognosis and the lack of targeted therapy continues to pose a challenge for management. Early diagnosis is imperative to reducing the disease burden of BLBC and the intricate dual functionality of miRNAs makes them a prime candidate for the production of an early detection model. Circulating miRNA expression signatures have been implicated as a means for the development of a potentially effective screening assay to detect BLBC via a liquid blood/plasma-based biopsy sample. Individualized and personalized targeted therapy for BLBC may considerably reduce the associated morbidity and mortality, and miRNAs stand as a potential means to achieve such. Drugs capable of effectively and safely silencing oncomiRs and/or potentiating tumor suppressive miRNAs associated with BLBC can substantially alter the present BLBC therapeutic landscape. The impact of miRNAs on BLBC tumorigenesis, tumor suppression, malignant transformation, and drug modulation, as highlighted in this review, is still subject to continued ongoing investigation. In conclusion, BLBC is an aggressive and complicated condition with no targeted therapy currently available and carries a significant burden to patients, but through previous and ongoing miRNA-based research, there is still hope.

## Figures and Tables

**Table 1:** Summary of the dual functional roles of microRNAs in breast cancer.

List of MicroRNA	Functional Role in Breast Cancer	Regulated Expression in Breast Cancer	Target(s)	Reference(s)
miR-10b	OncomiR	Upregulated	HOXD10	[70,71]
miR-21	OncomiR	Upregulated	PDCD4, PTEN	[72]
miR-155	OncomiR	Upregulated	SOCS1, TP53INP1, FOXO3, RhoA	[73]
miR-182-5p	OncomiR	Upregulated	CASP9	[74]
miR-191	OncomiR	Upregulated	DICER1	[61]
miR-375	OncomiR	Upregulated	RASD1	[75,76]
miR-425	OncomiR	Upregulated	DICER1	[61]
miR-125	Tumor suppressor	Downregulated	HuR, HER2, ETS1, Cyclin J, MEGF9	[73,76,77]
miR-145	Tumor suppressor	Downregulated	ARF6, fascin, JAM-A, C-MYC, ROCK1, CCNE2, RTKN, OCT4, MUC1, FSCN1, Rab27a	[78,79]
miR-200 family	Tumor suppressor	Differential	ZEB1, ZEB2	[73,76,80,81]
miR-205	Tumor suppressor	Downregulated	ZEB1, ZEB2, E2F1, LAMC1, KLF12, NFIB, AMOT, ERp29, HER3, CLCN3, SIP1, Ubc13, HMGB1/3, RunX2, ITGA5, VEGF-A, FGF2	[73,76,82]

Abbreviations: HOXD10—homeobox D10; PDCD4—programmed cell death 4; TPM1—tropomyosin-1; PTEN—phosphatase and TENsin homolog deleted; CDC25A—cell division cycle 25 A; RECK—reversion-inducing-cysteine-rich protein with kazal motifs; MASPIN—mammary serine protease inhibitor; TIMP3—tissue inhibitor of metalloproteinase-3; SOCS1—suppressor of cytokine signaling 1; TP53INP1—tumor protein 53-induced nuclear protein 1; FOXO3—forkhead box protein O3; RhoA—Ras homolog family member A; CASP9—caspase 9; DICER1—dicer 1, ribonuclease III; RASD1—dexamethasone-induced Ras-related protein 1; HuR—human antigen R; HER2—human epidermal growth factor receptor 2; ETS1—E26 transformation specific proto-oncogene 1; MEGF9—multiple epidermal growth factor like-domains 9; ARF6—adenosine diphosphate-ribosylation factor 6; JAM-A—junctional adhesion molecule A; C-MYC—cellular myelocytomatosis; ROCK1—rho-associated coiled-coil-containing protein kinase 1; CCNE2—cycline E2; RTKN—rhotekin; OCT4—octamer-binding transcription factor 4; MUC1—mucin 1; FSCN1—fascin actin-binding protein 1; ZEB1—zinc finger E-box binding homeobox 1; ZEB2—zinc finger E-box binding homeobox 2; E2F1—E2 promoter binding factor 1; LAMC1—laminin γ1 chain; KLF12—Krueppel-like factor 12; NFIB—nuclear factor 1 B-type; AMOT—angiomotin; ERp29—endoplasmic reticulum protein 29; HER3—human epidermal growth factor receptor 3; CLCN3—chloride voltage-gated channel 3; SIP1—Smad-interacting protein 1; Ubc13—ubiquitin-conjugating enzyme 13; HMGB1—high mobility group box 1; HMGB3—high mobility group box 3, RunX2—runt-related transcription factor 2; ITGA5—integrin alpha-5; VEGF-A—vascular endothelial growth factor A; FGF2—fibroblast growth factor 2.

**Table 2:** MicroRNA signature panels that distinguish BLBC/TNBC from other subtypes.

List of MicroRNA(s)	Sample Subtype	Significance of miRNA Expression	Reference(s)
miR-21	BLBC	Increased expression is highly associated with BLBC.	[91]
miR-101-3p miR-126-3p miR-126-5p miR-144-3p miR-144-5p miR-301a-3p miR-664b-5p	Basal-like TNBC	MiRNA panel differentiated healthy women from patients with basal-like TNBC with a sensitivity and specificity of 84% and 74%, respectively.	[84]
miR-205-5p miR-224-5p miR-375	BLBC	BLBC differentiated from non-basal TNBCs.	[92]
miR-146a miR-26a	TNBC	Significantly upregulated in TNBC relative to non-TNBC.	[93]
miR-146b miR-148a miR-200a miR-200b	TNBC	Downregulated specifically in TNBC.	[94]
ER status signature miRNAs: miR-135b miR-190 miR-217 miR-218 miR-299 miR-342 PR status signature miRNAs: miR-377 miR-520f-520c miR-520g miR-527-518a HER2 status signature miRNAs: miR-30e miR-181c miR-302c miR-376b miR-520d	TNBC	The miRNA signatures for ER, PR, and HER2 status were able to predict hormone receptor positivity/negativity with 100% accuracy. Incorporating all three panels together can allow for delineation of TNBC from other subtypes.	[95]
miR-210-3p miR-105-5p miR-767-5p miR-5683	TNBC	Upregulated in TNBC. Downregulated in TNBC.	[90]

Abbreviation: BLBC—basal-like breast cancer; TNBC—triple negative breast cancer; ER—estrogen receptor; PR—progesterone receptor; HER2—human epidermal growth factor receptor 2.

**Table 3:** MicroRNAs discerned as oncomiRs or tumor suppressors in BLBC/TNBC.

MicroRNA	Functional Role in BLBC	BLBC/TNBC	Differential Expression	Validated Target(s)	Reference(s)
miR-20a-5p	OncomiR	TNBC	Upregulated	RUNX3	[97]
miR-29a	OncomiR	TNBC	Upregulated	SUV420H2	[47,68]
miR-29b-3p	OncomiR	TNBC	Upregulated	TRAF3	[67]
miR-93	OncomiR	TNBC	Upregulated	LATS2, JAK1, STAT3, FBXL5, SOX4, EZH1, HMGA2	[87]
miR-105-5p	OncomiR	TNBC	Upregulated	AKT1, GRB2	[90,105]
miR-135b	OncomiR	BLBC	Upregulated	LATS2	[98,106]
miR-136	Tumor suppressor	TNBC	Downregulated	RASAL2	[107]
miR-140	Tumor suppressor	BLBC	Downregulated	SOX9, ALDH1, TRIM28	[102,108,109]
miR-146a	OncomiR	TNBC	Upregulated	BRCA1, NRP2	[107,110]
miR-146a-5p	Tumor suppressor	TNBC	Downregulated	SOX5	[111]
miR-181a	OncomiR	TNBC	Upregulated	BIM, ATM, BAX	[107]
miR-181b	OncomiR	TNBC	Upregulated	BIM, ATM	[107]
miR-182	OncomiR	TNBC	Upregulated	PFN1, RECK, FOXF2	[107,112,113]
miR-182-3p	Tumor suppressor	TNBC	Downregulated	TRF2	[104]
miR-200a	Tumor suppressor	BLBC and TNBC	Downregulated	EPHA2, ZEB1, ZEB2	[94,107]
miR-200b	Tumor suppressor	BLBC and TNBC	Downregulated	PKCα, ZEB1, ZEB2	[94,107,114]
miR-210-3p	OncomiR	TNBC	Upregulated	GPD1L, CYGB	[90,115]
miR-221	OncomiR	BLBC and TNBC	Upregulated	ANXA3, PTEN, TRPS1	[107,116,117,118]
miR-222	OncomiR	BLBC	Upregulated	ANXA3, PTEN, TRPS1	[116,117,118]
miR-296-5p	Tumor suppressor	BLBC	Downregulated	hTERT	[119]
miR-342	OncomiR	BLBC and TNBC	Upregulated	ID4	[107,120]
miR-512-5p	Tumor suppressor	BLBC	Downregulated	hTERT	[119]
miR-767-5p	OncomiR	TNBC	Upregulated	SOCS2	[90,121]
miR-3940-3p	OncomiR	TNBC	Upregulated	KLLN	[122]

Abbreviations: BLBC—basal-like breast cancer; TNBC—triple-negative breast cancer; RUNX3—runt-related transcription factor 3; SUV420H2—suppressor of variegation 4–20 homolog 2; TRAF3—tumor necrosis factor receptor associated factor 3; LATS2—large tumor suppressor kinase 2; JAK1—Janus kinase 1; STAT3—signal transducer and activator of transcription 3; FBXL5—F-box and leucine-rich repeat protein 5; SOX4—sex-determining region Y-related high-mobility group box 4; EZH1—enhancer of zeste 1; HMGA2—High-mobility group AT-hook 2; AKT1—Ak strain transforming; GRB2—Growth Factor Receptor-bound protein 2; RASAL2—RAS protein activator like 2; SOX9—sex-determining region Y-related high-mobility group box 9; ALDH1—aldehyde dehydrogenase 1; TRIM28—tripartite motif-containing 28; BRCA1—breast cancer gene 1; NRP2—neuropilin 2; SOX5—sex-determining region Y-related high-mobility group box 5; BIM—Bcl-2-like protein 11; ATM-ataxia telangiectasia–mutated; BAX—Bcl-2-associated X protein; PFN1—profilin 1; RECK—reversion-inducing-cysteine-rich protein with kazal motifs; FOXF2—forkhead box F2; TRF2—telomeric repeat-binding factor 2; EPHA2—ephrin type-A receptor 2; ZEB1—zinc finger E-box binding homeobox 1; ZEB2—zinc finger E-box binding homeobox 2; PKCα—protein kinase C alpha; GPD1L—glycerol-3-phosphate dehydrogenase 1-like; CYGB—cytoglobin; ANXA3—annexin A3; PTEN-phosphatase and TENsin homolog deleted; TRPS1—transcriptional repressor GATA binding 1; hTERT—human telomerase reverse transcriptase; ID4—Inhibitor Of DNA Binding 4; SOCS2—suppressor of cytokine signaling 2; KLLN—Killin, p53-regulated DNA replication inhibitor.

**Table 4:** MiRNAs signature panels and their prognostic implication in BLBC/TNBC.

MicroRNAs in Panel	BLBC/TNBC	Prognostic Implication	Reference(s)
miR-155	TNBC	Significantly associated with overall survival (*p* < 0.01).	[123,125,126]
miR-30a-5p miR-30a-3p miR-30c-5p	Basal-like TNBC and TNBC	Dysregulation of miR-30 expression was seen to impede the recurrence free survival.	[92,93,94,95,96,97,98,99,100,101,102,103,104,105,106,107,108,109,110,111,112,113,114,115,116,117,118,119,120,121,122,123]
miR-30a-3p miR-30a-5p miR-199a-5p miR-203b-5p miR-324-5p let-7d-3p	Basal-like TNBC and TNBC	Panel signatures were significantly associated with decreased overall survival (OS).	[92]
miR-30a-3p miR-30a-5p miR-30c-5p miR-128-3p let-7d-3p	Basal-like TNBC and TNBC	Panel signatures were significantly associated with decreased relapse-free survival (RFS).	[92]
miR-95-3p	Basal-like TNBC and TNBC	Elevated expression of miR-95-3p in patients treated with anthracycline chemotherapeutics correlates with a diminished overall survival (*p* = 0.03) and relapse-free survival (*p* < 0.01).	[92]
miR-146a miR-26a miR-10b miR-153	TNBC	Expression of miR-146a and miR-26a is greater in node-negative TNBC relative to node-positive TNBC (*p* = 0.04 and 0.01, respectively), whereas miR-10b showed the inverse relationship (*p* = 0.05).	[93]
miR-1	TNBC	MALAT1, a long noncoding RNA upregulated in TNBC, possesses known oncogenic function and has a reciprocal relationship with miR-1 expression. Excessive MALAT1 expression is associated with poor overall survival relative to low.	[127]
miR-27a miR-30e miR-155 miR-493	BLBC	Panel signatures were significantly associated with median overall survival (*p* < 0.01).	[125]
miR-10b miR-21 miR-31 miR-125b miR-130a-3p miR-155 miR-181a miR-181b miR-451a	TNBC	The listed miRNAs were all found to be dysregulated in TNBC and were found to be associated with pathways of chemotherapy resistance.	[89]
miR-190a miR-200b-3p miR-512-5p	TNBC	A panel incorporating expression levels of the three listed miRNAs was found to be associated with favorable chemotherapy activity (*p* = 0.06).	[128]
miR-125b miR-655 miR-421 miR-16 miR-374a miR-374b miR-497	TNBC	Panel signatures were significantly associated with median distant disease-free survival (DFS) (*p* < 0.01).	[126]
miR-16 miR-155 miR-374a miR-125b	TNBC	Panel signatures were significantly associated with overall survival (*p* = 0.05).	[126]
miR-148a miR-200b	TNBC and BLBC	Both miRNAs modulate the E2F pathway, whose function is associated with poor prognosis, and both are significantly under expressed in TNBC.	[94]

Abbreviations: BLBC—basal-like breast cancer; TNBC—triple-negative breast cancer; MALAT1—metastasis associated in lung adenocarcinoma transcript 1.
